# Adrenal Masses in a Moroccan Tertiary Center: A 14-Year Retrospective Study of 200 Patients

**DOI:** 10.7759/cureus.116105

**Published:** 2026-09-11

**Authors:** Meryem El Adla, Zineb Elazime, Mohammed Amine Essafi, Hayat Aynaou, Houda Salhi

**Affiliations:** 1 Department of Endocrinology, Diabetology, Metabolic Diseases, and Nutrition, Hassan II University Hospital, Fez, MAR

**Keywords:** adrenal incidentaloma, adrenal masses, adrenal tumors, morocco, tertiary care center

## Abstract

Objectives

Adrenal masses are increasingly detected and encompass a broad spectrum of clinical presentations and pathological diagnoses. This study aimed to describe the epidemiological, clinical, paraclinical, etiological, and therapeutic profiles of patients with adrenal masses.

Materials and methods

This single-center retrospective descriptive study included consecutive eligible patients hospitalized for adrenal masses at the Department of Endocrinology, Diabetology, Metabolic Diseases, and Nutrition of Hassan II University Hospital in Fez, Morocco, between January 2009 and January 2023. Data were retrospectively collected from medical records between January and February 2023.

Results

A total of 200 patients were included, with a mean age of 49 years and a predominance of female patients (n = 142, 71%). The most common circumstance of discovery was adrenal incidentaloma (n = 110, 55%). The etiological diagnoses were non-secreting adenoma (n = 73, 36.5%), pheochromocytoma (n = 33, 16.5%), Conn’s adenoma (n = 22, 11%), cortisol-secreting adenoma (n = 20, 10%), adrenocortical carcinoma (n = 18, 9%), adrenal metastases (n = 13, 6.5%), adrenal cysts (n = 7, 3.5%), lymphoma (n = 5, 2.5%), myelolipoma (n = 4, 2%), ganglioneuroma (n = 2, 1%), schwannoma (n = 2, 1%), and oncocytic adenoma (n = 1, 0.5%). Surgical treatment was performed in 80 patients (40%), documented clinical and biological surveillance was performed in 43 patients (21.5%), and 10 patients (5%) received palliative treatment.

Conclusion

This study demonstrates the diversity of adrenal mass etiologies and clinical presentations and highlights the importance of a multidisciplinary diagnostic and therapeutic approach.

## Introduction

Adrenal masses vary in embryological origin, endocrine activity, and malignant potential. Their reported prevalence is approximately 2.1% and is increasing with the widespread use of imaging modalities [1]. The diagnostic approach aims to distinguish benign from malignant lesions and functioning from non-functioning tumors [2].

The initial evaluation relies on clinical assessment and modern biochemical testing. Imaging facilitates tumor localization and the assessment of malignant features. Secreting adrenal masses represent approximately 50% of cases, while malignant lesions account for around 22% [3]. Secreting tumors often require surgical treatment, whereas benign non-secreting masses usually require surveillance [1].

To our knowledge, no study has been previously conducted in Morocco to evaluate the epidemiological, clinical, paraclinical, etiological, and therapeutic profiles of patients with adrenal masses. The objective of this study was to characterize a large cohort of patients managed in a Moroccan tertiary center over a 14-year period, so as to provide data specific to the region that may help in clinical decision-making, multidisciplinary management, and the application of international recommendations in North African healthcare settings.

## Materials and methods

This was a single-center retrospective descriptive study conducted at the Department of Endocrinology, Diabetology, Metabolic Diseases, and Nutrition of Hassan II University Hospital in Fez, Morocco. The study included patients managed for adrenal masses between January 2009 and January 2023. Data were retrospectively collected from medical records between January and February 2023.

Consecutive sampling was used. All patients hospitalized for an adrenal mass during the study period who had sufficiently complete clinical, biochemical, radiological, therapeutic, and outcome data and who consented to the use of their clinical data for research purposes were included. Patients with incomplete records, patients lost to follow-up before the required outcome information could be obtained, and patients who did not consent to the use of their data were excluded. The total number of records initially screened and the number excluded because of incomplete data were not retained in the final anonymized study database and could not be retrospectively recovered.

Collected variables included sociodemographic, clinical, biological (urinary or plasma metanephrines, cortisol rhythm, urinary free cortisol, the 1-mg overnight dexamethasone suppression test, and the aldosterone-to-renin ratio), radiological, etiological, therapeutic, histopathological, and follow-up data. Because this was a descriptive study without causal or comparative analyses, no statistical adjustment for confounders was performed. Potential sources of bias included age, sex, comorbidities, referral to a tertiary center, the retrospective design, incomplete documentation, and changes in diagnostic practices during the 14-year period.

Hormonal evaluation

Hormonal evaluation included assessment of cortisol, catecholamine, and aldosterone secretion, as clinically indicated. Autonomous cortisol secretion was evaluated primarily using the 1-mg overnight dexamethasone suppression test. A morning serum cortisol concentration ≤50 nmol/L (≤1.8 µg/dL) after dexamethasone was considered to exclude autonomous cortisol secretion, whereas a value >50 nmol/L (>1.8 µg/dL) was considered abnormal in the context of an adrenal incidentaloma. Additional evaluation included the cortisol rhythm and urinary free cortisol when clinically indicated.

Catecholamine excess was assessed using plasma-free metanephrines and/or urinary fractionated metanephrines. Values above the laboratory-specific upper reference limit were considered abnormal. Mild elevations were reassessed under appropriate sampling conditions when clinically indicated, whereas values approximately three times the upper reference limit were considered strongly suggestive of pheochromocytoma.

Primary aldosteronism was screened for in patients with hypertension and/or hypokalemia using plasma aldosterone, direct renin concentration, and the aldosterone-to-renin ratio. The criteria used included a plasma aldosterone concentration ≥277 pmol/L, a direct renin concentration ≤8.2 mIU/L, and an aldosterone-to-renin ratio ≥70 pmol/mIU. These findings were considered suggestive of primary aldosteronism and were interpreted in conjunction with the clinical context and subsequent investigations.

Because the analytical techniques used to measure cortisol, aldosterone, renin, and plasma and urinary metanephrines evolved during the 14-year study period, each result was interpreted according to the assay method, measurement units, and laboratory reference ranges applicable at the time of testing. Hyperaldosteronism was identified by an elevated aldosterone concentration combined with suppressed renin and an aldosterone-to-renin ratio exceeding the applicable laboratory threshold. Catecholamine excess was defined by plasma and/or urinary metanephrine concentrations above the applicable upper reference limit. Hypercortisolism was assessed according to the test performed and its contemporaneous reference threshold, particularly the failure of cortisol suppression following dexamethasone.

Radiological assessment

Adrenal lesions were assessed using non-contrast attenuation, size, homogeneity, margins, contrast washout when available, local invasion, and interval growth. Absolute and relative washout could be calculated when the CT protocol included unenhanced, portal venous, and delayed acquisitions.

Absolute washout was calculated as:

\( \text{Absolute Washout} = \frac{\text{Enhanced Attenuation} - \text{Delayed Attenuation}} {\text{Enhanced Attenuation} - \text{Unenhanced Attenuation}} \times 100 \)

Relative washout was calculated as:

\( \text{Relative Washout} = \frac{\text{Enhanced Attenuation} - \text{Delayed Attenuation}} {\text{Enhanced Attenuation}} \times 100 \)

An absolute washout ≥60% or a relative washout ≥40% was considered suggestive of an adenoma.

Lesions were considered indeterminate or suspicious when they had an unenhanced attenuation >10 HU, washout values not consistent with an adenoma, a diameter ≥4 cm, or unfavorable morphological features, including heterogeneity, irregular margins, necrosis, hemorrhage, local invasion, or growth during follow-up. When all acquisition phases were unavailable, washout could not be calculated, and radiological assessment was based on the remaining CT characteristics.

Follow-up

The frequency and duration of follow-up were not uniform because they were individualized according to the date of diagnosis, treatment strategy, clinical status, and subsequent disease course. Individual follow-up duration was calculated from the date of diagnosis to the last documented consultation. Consequently, patients diagnosed earlier could be followed for several years, whereas those diagnosed near the end of the inclusion period had shorter follow-up.

After surgery, patients were generally assessed approximately one week, one month, and three months after the procedure, with subsequent visits progressively spaced according to their clinical and biochemical course. Patients managed non-operatively or considered clinically stable were generally assessed every three to six months. Surveillance was based on documented clinical evaluation, relevant biochemical measurements, and radiological reassessment when indicated.

Recurrence was defined as the reappearance of disease after initial treatment or after a documented period without clinically, biochemically, or radiologically detectable disease. It was assessed through clinical examination, relevant hormonal testing, and imaging, with histopathological confirmation when tissue was available.

Multidisciplinary assessment

Complex cases and cases requiring specialized therapeutic decisions were discussed at multidisciplinary meetings. Depending on the characteristics of each case, these meetings involved endocrinologists, visceral surgeons, medical oncologists, radiation oncologists, radiologists, gastroenterologists, otorhinolaryngologists, and gynecologists. Diagnostic, therapeutic, and follow-up strategies were determined collaboratively and individualized for each patient.

Management definitions

Conservative management was defined as the absence of immediate surgical or oncological treatment. It consisted of clinical, biochemical, and radiological surveillance, with medical treatment of hormonal manifestations and comorbidities when required. This strategy was used primarily for lesions considered benign or stable, non-functioning tumors, and patients for whom the anticipated benefits of intervention did not outweigh its risks.

Palliative treatment was defined as treatment provided to patients with advanced or metastatic disease that was not amenable to curative therapy. Management was individualized by the medical oncology and radiation oncology teams according to the protocols applicable at the time. It could include systemic treatment, analgesic or locoregional radiotherapy, and supportive care for pain, symptoms of disease progression, and endocrine complications. Its objectives were to control disease progression when feasible and to preserve or improve quality of life.

No formal sample size calculation was performed because all consecutive eligible and consenting patients available during the predefined study period were included. Statistical analysis was performed using SPSS Statistics for Windows, version 16.0 (SPSS Inc., Chicago, Ill., USA). Categorical variables were summarized as counts and percentages (n (%)), and continuous variables were summarized using the mean and range. No group comparisons or inferential statistical analyses were performed; therefore, no statistical tests, test statistics (t, χ², or F), or p-values were applicable. The study was approved in December 2022 by the Ethics Committee of Hassan II University Hospital in Fez. Informed consent for the use of clinical data for research purposes was obtained from all included patients. All data were anonymized, and patient confidentiality was maintained.

## Results

A total of 200 patients were included. Their sociodemographic and clinical characteristics are shown in Table 1. The mean age was 49 years (range, 18-77 years), and 142 patients (71%) were women.

**Table 1:** Sociodemographic and clinical characteristics (N = 200)

Characteristic	n (%) or value
Age, mean years	49 (18–77)
Women	142 (71%)
Men	58 (29%)
Hypertension	80 (40%)
Diabetes mellitus	45 (22.5%)
Hypokalemia	23 (11.5%)
Incidental discovery	110 (55%)
Unilateral adrenal mass	154 (77%)
Bilateral adrenal masses	46 (23%)

Hormonal and radiological findings are presented in Table 2. Hormonal hypersecretion was identified in 88 patients (44%), and CT was performed in 197 patients (98.5%).

**Table 2:** Hormonal and radiological findings (N = 200)

Characteristic	n (%) or value
Hormonal hypersecretion	88 (44%)
Hypercortisolism	33 (16.5%)
Catecholamine excess	33 (16.5%)
Hyperaldosteronism	22 (11%)
CT performed	197 (98.5%)
Mean tumor size	37 mm
Non-contrast CT attenuation ≤10 HU	106 (53%)

Etiological diagnoses are summarized in Table 3 and illustrated in Figure 1. The most common diagnosis was non-secreting adenoma, observed in 73 patients (36.5%), followed by pheochromocytoma in 33 patients (16.5%). Eight of the 33 patients with pheochromocytoma had bilateral disease; six of these eight patients had multiple endocrine neoplasia type 2 (MEN2), and two had sporadic disease.

**Table 3:** Etiological diagnoses

Etiological diagnosis	n (%)
Non-secreting adenoma	73 (36.5%)
Pheochromocytoma	33 (16.5%)
Conn’s adenoma	22 (11%)
Cortisol-secreting adenoma	20 (10%)
Adrenocortical carcinoma	18 (9%)
Adrenal metastasis	13 (6.5%)
Adrenal cyst	7 (3.5%)
Lymphoma	5 (2.5%)
Myelolipoma	4 (2%)
Ganglioneuroma	2 (1%)
Schwannoma	2 (1%)
Oncocytic adenoma	1 (0.5%)
Bilateral pheochromocytoma (subset)	8/33 (24.2%)
MEN2-associated bilateral disease (subset)	6/8 (75%)
Sporadic bilateral disease (subset)	2/8 (25%)
Total	200 (100%)

**Figure 1:**
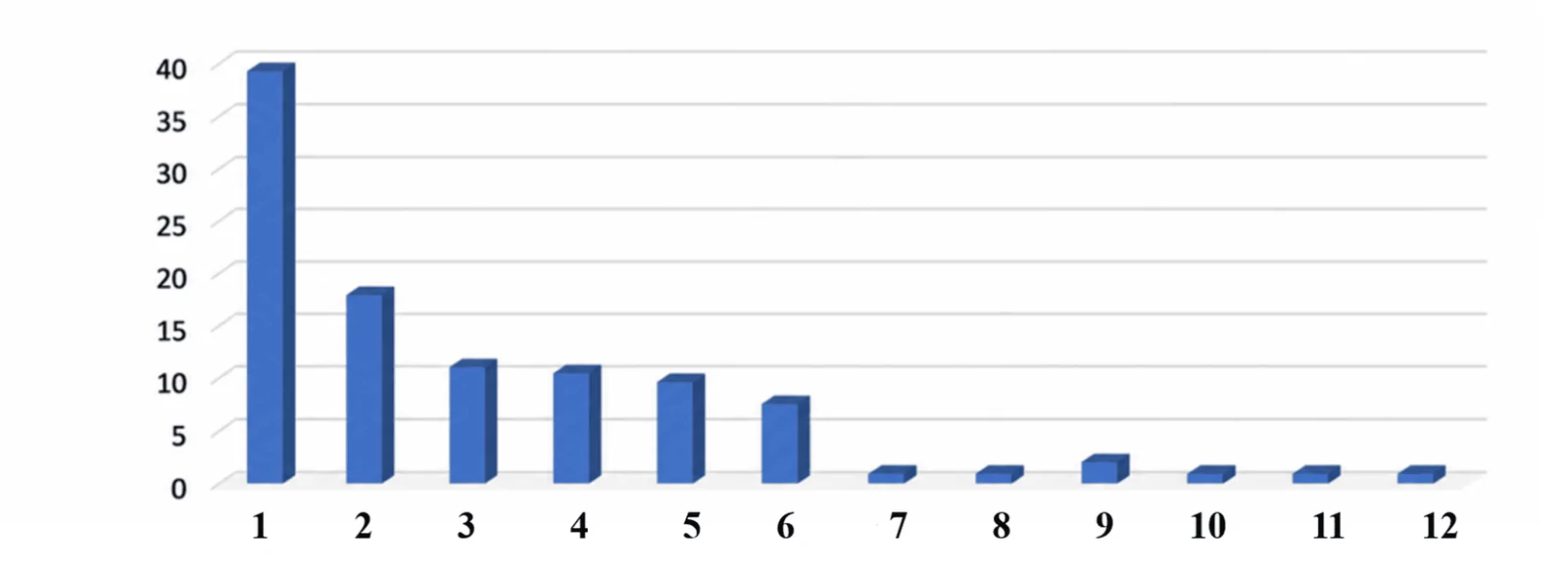
Frequency of adrenal masses in our series 1. Non-secreting adenoma; 2. Pheochromocytoma; 3. Conn’s adenoma; 4. Cortisol-secreting adenoma; 5. Adrenocortical carcinoma; 6. Adrenal metastasis; 7. Adrenal cyst; 8. Lymphoma; 9. Myelolipoma; 10. Ganglioneuroma; 11. Schwannoma; 12. Oncocytic adenoma.

Therapeutic and follow-up findings are presented in Table 4. Surgical treatment was performed in 80 patients (40%), documented clinical and biological surveillance was performed in 43 patients (21.5%), and palliative treatment was administered to 10 patients (5%). Seven patients (3.5%) experienced recurrence during follow-up. Of the seven documented recurrences, three were identified during postoperative follow-up, whereas four occurred after a documented period without clinically, biochemically, or radiologically detectable disease. The mean follow-up duration among patients with documented follow-up was five years. Most of these patients remained under regular outpatient follow-up at the time of the last available assessment. Seven patients (3.5%) experienced recurrence during follow-up. Of these, three recurrences were identified during postoperative surveillance, whereas four occurred after a documented period without clinically, biochemically, or radiologically detectable disease.

**Table 4:** Therapeutic and evolutionary findings

Finding	n/N (%)
Surgical treatment	80/200 (40%)
Secreting tumors among operated patients	74/80 (92.5%)
Malignant tumors among operated patients	6/80 (7.5%)
Unilateral adrenalectomy among operated patients	56/80 (70%)
Bilateral adrenalectomy among operated patients	24/80 (30%)
Conservative management of non-secreting adenomas	63/73 (86.3%)
Conservative management of adrenal cysts	5/7 (71.4%)
Documented clinical and biological surveillance	43/200 (21.5%)
Palliative treatment	10/200 (5%)
Medically managed bilateral Conn’s adenoma	2/200 (1%)
Recurrence	7/200 (3.5%)
Pheochromocytoma recurrence with bone metastases	3/200 (1.5%)
Malignant Cushing syndrome recurrence	4/200 (2%)

The 103 histopathological records available in the study database are summarized in Table 5. These records comprised surgical specimens, biopsy specimens, and external pathology reports; therefore, they did not correspond exclusively to the 80 adrenalectomies performed at our center.

**Table 5:** Histopathological diagnoses recorded in the study database (N = 103) records

Histopathological diagnosis	n (%)
Pheochromocytoma	33 (32.0%)
Non-secreting adenoma	11 (10.7%)
Conn’s adenoma	20 (19.4%)
Cortisol-secreting adenoma	20 (19.4%)
Adrenocortical carcinoma	7 (6.8%)
Adrenal metastasis	1 (1.0%)
Adrenal cyst	2 (1.9%)
Myelolipoma	4 (3.9%)
Ganglioneuroma	2 (1.9%)
Schwannoma	2 (1.9%)
Oncocytic adenoma	1 (1.0%)
Total	103 (100%)

Representative imaging findings of bilateral Conn’s adenomas, adrenocortical carcinoma, and myelolipoma are shown in Figures 2-4, respectively. 

**Figure 2:**
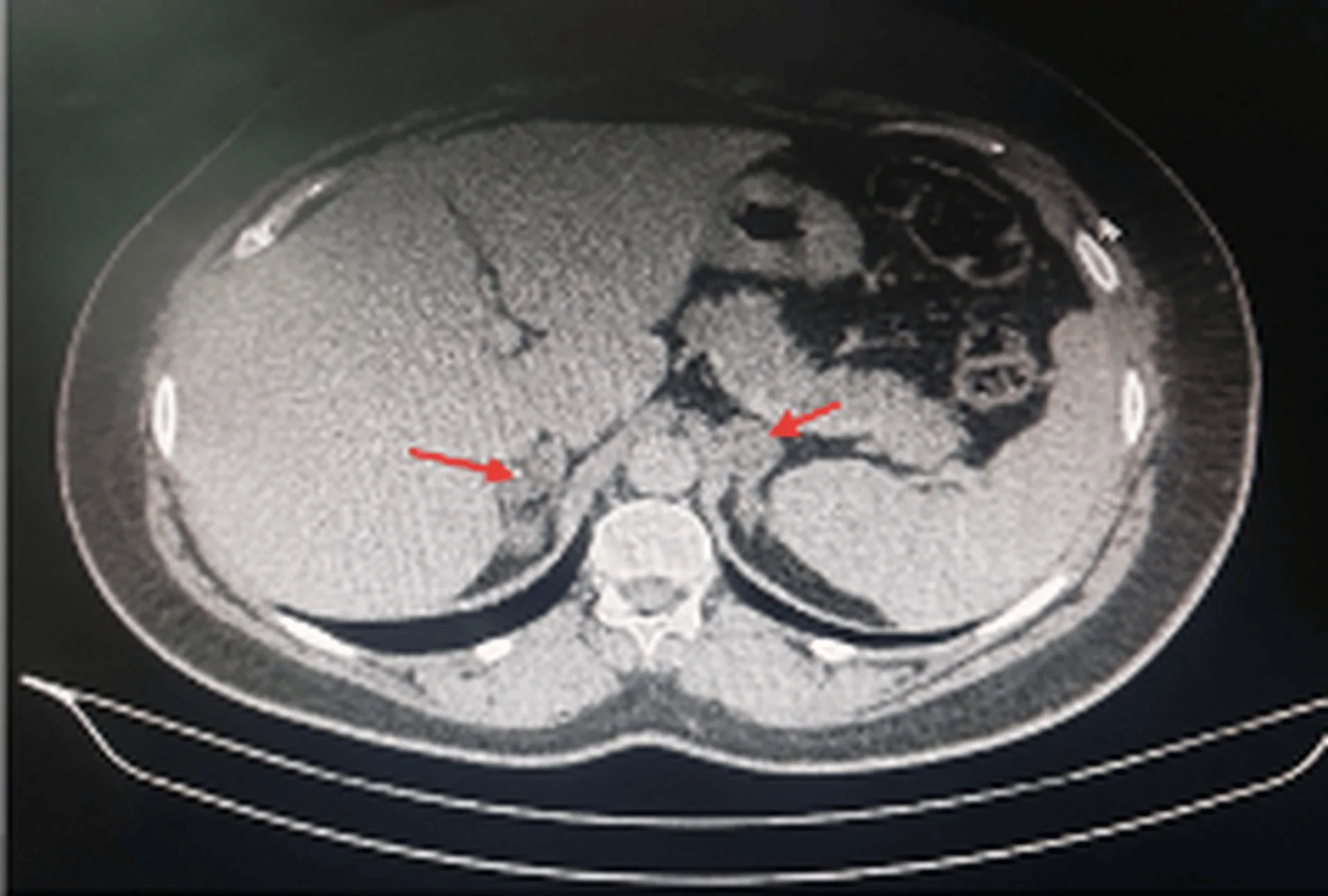
Axial CT image showing bilateral hypodense adrenal lesions consistent with bilateral Conn's adenomas Image courtesy of the Department of Radiology, Hassan II University Hospital, Fez, Morocco.

**Figure 3:**
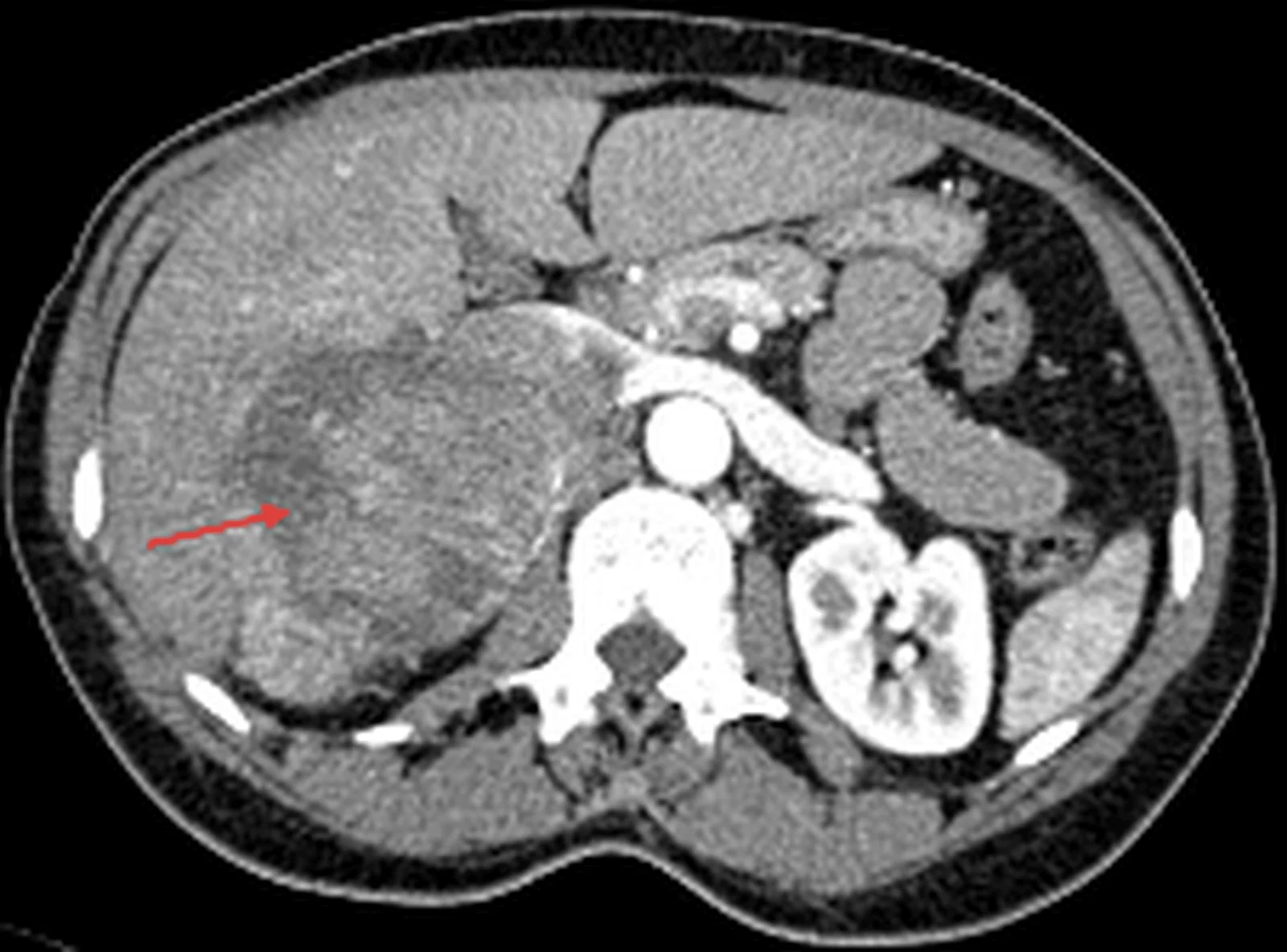
Contrast-enhanced axial CT image showing a large heterogeneous right adrenocortical carcinoma Image courtesy of the Department of Radiology, Hassan II University Hospital, Fez, Morocco.

**Figure 4:**
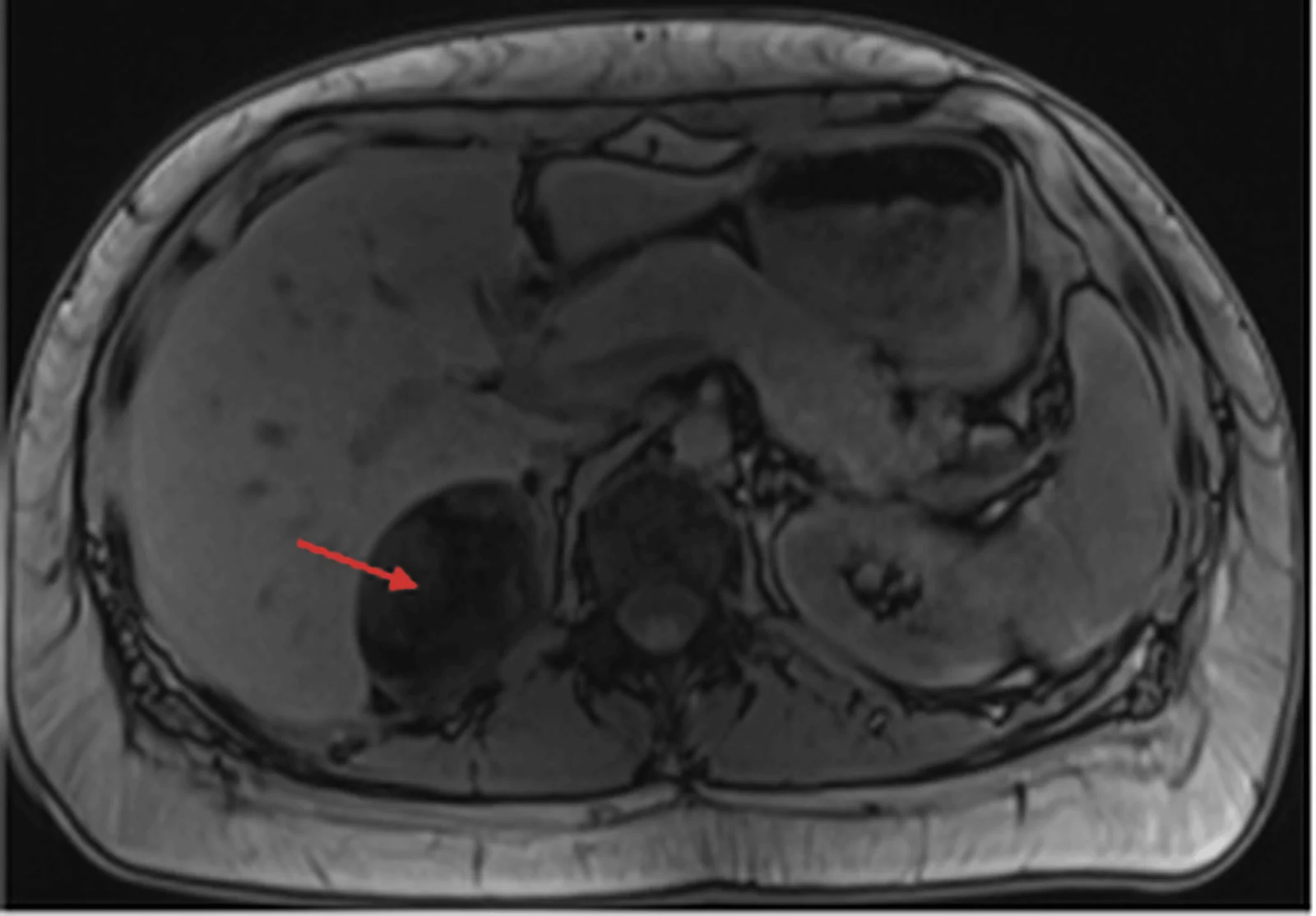
Axial MRI showing a heterogeneous right adrenal nodule measuring 64 x 44 x 54 mm, with macroscopic fat T1/T2 hyperintensity and an enhancing tissue component after gadolinium administration Image courtesy of the Department of Radiology, Hassan II University Hospital, Fez, Morocco.

## Discussion

Sociodemographic data

In our series, the mean age at diagnosis was 49 years, consistent with the literature showing a peak incidence of adrenal masses in the fifth and sixth decades of life [1,3]. The increased detection of adrenal masses in this age group is largely attributable to the growing use of imaging [1].

The European Society of Endocrinology (ESE) guidelines confirm that adrenal incidentalomas are more frequently identified in women, although malignant lesions and metastases may show a more balanced sex distribution [1]. In our series, women predominated (n = 142, 71%). This may reflect biological predisposition, healthcare-seeking behavior, and referral patterns, as tertiary endocrine centers often receive a higher proportion of hormonally active tumors, particularly cortisol- and aldosterone-secreting adenomas, which are more frequently diagnosed in women [1,4].

Clinical and paraclinical data

According to the 2023 European Society of Endocrinology/European Network for the Study of Adrenal Tumors (ESE/ENSAT) guidelines, most newly diagnosed adrenal masses are incidentalomas detected during imaging for non-adrenal indications [1]. This is consistent with our finding that 110 patients (55%) had adrenal masses discovered incidentally and with international data indicating that more than half of adrenal tumors are incidental findings [3].

Unilateral masses predominated in our study (n = 154 (77%)), consistent with previous imaging studies showing that bilateral adrenal masses are less common. Bilateral disease may be associated with specific etiologies, including metastatic disease, congenital adrenal hyperplasia, macronodular adrenal hyperplasia, and bilateral pheochromocytoma [5,6].

In our series, hormonal hypersecretion was identified in 88 patients (44%), including hypercortisolism in 33 patients (16.5%), catecholamine excess in 33 patients (16.5%), and hyperaldosteronism in 22 patients (11%). This proportion is slightly higher than that reported in the literature, in which non-functioning adenomas predominate. This discrepancy may be explained by referral bias at our tertiary center, where hormonally active lesions are more likely to be hospitalized and evaluated [1,3].

Hypercortisolism (n = 33 (16.5%)) was one of the most frequent patterns of hormonal hypersecretion. Recent ESE guidelines define mild autonomous cortisol secretion (MACS) using the 1-mg overnight dexamethasone suppression test, with a post-dexamethasone serum cortisol concentration greater than 50 nmol/L (>1.8 micrograms/dL) indicating increased risk [1]. Even mild cortisol excess is associated with greater cardiometabolic risk. Hypertension and diabetes were common in our cohort (n = 80 (40%) and n = 45 (22.5%), respectively), underscoring the importance of systematic endocrine evaluation in patients with adrenal masses [1].

Primary aldosteronism was found in 22 patients (11%). The 2023 ESE/ENSAT guideline recommends assessment using the aldosterone-to-renin ratio in patients with hypertension or unexplained hypokalemia [1]. In addition, hypertension and hypokalemia (n = 23 (11.5%) in our study) should systematically prompt biochemical screening, as emphasized in current recommendations [1,4].

Pheochromocytoma was diagnosed in 33 patients (16.5%), a proportion higher than that generally reported in unselected cohorts but consistent with the referral pattern of a tertiary center. Measurement of plasma-free or urinary fractionated metanephrines remains central to diagnosis [1,2]. Current guidelines recommend excluding pheochromocytoma in adrenal masses that are not clearly benign on imaging, particularly lesions with an unenhanced CT attenuation greater than 10 HU, because of the risk of life-threatening complications [1].

A non-contrast CT attenuation of 10 HU or less was observed in 106 patients (53%). Current guidelines consider homogeneous lesions with an attenuation of 10 HU or less to be benign adenomas that require no further imaging follow-up, regardless of size [1]. In contrast, lesions with an attenuation greater than 20 HU or heterogeneous features require multidisciplinary discussion because of their increased risk of malignancy [1,5].

Etiological data

In our series, non-secreting adenomas were the most frequent etiology (n = 73 (36.5%)), consistent with international studies where they account for the majority of adrenal incidentalomas [1,3]. The diagnosis is based on imaging criteria (low attenuation, homogeneous appearance, rapid washout) and absence of hormonal hypersecretion [5].

Pheochromocytoma was the second most frequent etiology (n = 33, 16.5%). Eight patients had bilateral disease, including six MEN2-associated cases. Genetic syndromes account for a substantial proportion of bilateral pheochromocytomas, and guidelines recommend genetic counseling for patients with bilateral, multifocal, or early-onset disease [1,2]. Genetic testing was not systematically performed in all patients with bilateral pheochromocytoma. Evaluation for MEN2 was undertaken primarily in patients with suggestive clinical features or a relevant family history. Therefore, the six identified MEN2-associated cases may underestimate the actual prevalence of hereditary disease in this subgroup.

Conn’s adenoma was the third most frequent etiology (n = 22 (11%)). Primary aldosteronism remains underdiagnosed in clinical practice [4]; therefore, our rate may reflect more active screening among hypertensive patients.

Adrenocortical carcinoma (ACC) occurred in 18 patients (9%). Although rare, ACC remains clinically significant because of its aggressive nature. The ESE/ENSAT guidelines highlight the importance of suspicious imaging characteristics (size >4 cm, irregular margins, and high attenuation) and early surgical management in specialized centers [1,7]. The relatively high proportion of ACC in our series may reflect referral of advanced cases to our tertiary hospital.

Adrenal metastases and primary adrenal lymphoma represented 13 patients (6.5%) and five patients (2.5%), respectively. These etiologies are often associated with bilateral involvement and require comprehensive oncological evaluation [8,9]. Their presence reinforces the importance of considering non-endocrine causes in the differential diagnosis of adrenal masses.

Adrenal cysts occurred in seven patients (3.5%) and were mostly benign and managed conservatively. Cystic adrenal lesions are often incidental findings and generally require surgery only when symptomatic or suspicious for malignancy [10].

Myelolipomas occurred in four patients (2%). These benign tumors are composed of mature adipose and hematopoietic tissue. They are typically non-functioning and are characterized radiologically by macroscopic fat on CT, allowing confident diagnosis without surgery in most cases [5].

Ganglioneuroma and schwannoma each occurred in two patients (1%). These rare neural crest-derived tumors are usually non-secreting and are often diagnosed postoperatively by histopathological examination because of nonspecific imaging findings.

Oncocytic adenoma occurred in one patient (0.5%). This is a rare adrenal cortical tumor variant. Although most are benign, some may have uncertain malignant potential and require careful pathological evaluation.

Therapeutic and evolutionary aspects

Surgical treatment was performed in 80 patients (40%), mainly for functioning tumors and lesions suspected of malignancy. ESE recommendations support adrenalectomy for hormone-secreting tumors, lesions suspicious for malignancy, and selected cases of MACS with clinically relevant comorbidities. Preoperative alpha-adrenergic blockade is essential in patients with pheochromocytoma to reduce perioperative risk [2] and was used in our cohort. Conservative management was adopted for non-functioning adenomas smaller than 4 cm with benign imaging features. Current ESE guidance does not recommend routine repeat imaging for homogeneous lesions with an unenhanced attenuation of 10 HU or less [1].

Recurrence occurred in seven patients (3.5%) with malignant pheochromocytoma or ACC. Long-term follow-up is crucial in these patients, as highlighted by ENSAT recommendations, particularly in the presence of high-risk histological features [1,7].

Clinical recommendations based on current guidelines

Based on our findings and international recommendations [1], we emphasize systematic hormonal evaluation of adrenal masses using the 1-mg overnight dexamethasone suppression test, plasma-free or urinary fractionated metanephrines, and the aldosterone-to-renin ratio in patients with hypertension. Initial non-contrast CT should assess lesion homogeneity and attenuation. An attenuation of 10 HU or less supports a benign diagnosis, whereas a size greater than 4 cm, an attenuation greater than 20 HU, or heterogeneous features should prompt multidisciplinary review. Surgery should be considered for functioning tumors and lesions suspected of malignancy, while follow-up should be individualized to avoid unnecessary imaging of clearly benign adenomas.

Strengths and limitations

This study represents one of the largest single-center series of adrenal masses reported in Morocco, including 200 patients managed over a 14-year period. Its strengths include the size and duration of the cohort, the availability of hormonal and radiological characterization, and the inclusion of therapeutic and follow-up data permitting the description of management strategies and documented recurrences.

Several limitations should be considered. The retrospective design exposed the study to selection bias, incomplete documentation, and heterogeneity in the available data. As our institution is a tertiary referral center, complex, hormonally active, and potentially malignant lesions were likely overrepresented, which may explain the higher proportion of functioning tumors compared with population-based cohorts [1,3]. The magnitude of this referral bias could not be quantified; therefore, the etiological distribution observed in our cohort may not be generalizable to patients managed in primary or secondary care settings.

The 103 available histopathological records included adrenalectomy specimens obtained at our center, biopsy specimens, and external pathology reports. Because these specimens were evaluated by different pathologists and institutions over a 14-year period, variations in pathological assessment and diagnostic criteria may have affected diagnostic consistency. Genetic testing was not systematically available for all eligible patients. In addition, diagnostic methods, hormonal assays, imaging protocols, pathological criteria, and therapeutic recommendations evolved during the study period, but temporal trends in diagnosis and management were not analyzed separately.

The exclusively descriptive analysis did not permit comparisons between functioning and non-functioning tumors or between benign and malignant lesions and could not identify factors associated with malignancy, hormonal activity, treatment selection, or recurrence. Surgical complications, perioperative mortality, and other postoperative outcomes were not systematically analyzed. The final anonymized database did not retain the total number of records initially screened or the number excluded because of incomplete data, preventing quantitative assessment of the associated selection bias. Although the mean follow-up duration among patients with documented follow-up was five years, follow-up schedules were individualized, and complete follow-up information was not uniformly available for the entire cohort. These factors limit the interpretation of the seven documented recurrences, which should be considered a descriptive proportion rather than a formal recurrence rate.

Despite these limitations, this study provides valuable regional data on the presentation and management of adrenal masses and may inform the implementation of international recommendations in a North African tertiary-care setting.

## Conclusions

Adrenal masses represent a heterogeneous group of tumors with variable clinical, hormonal, and radiological presentations. In this 14-year single-center experience, non-secreting adenomas were the most frequent etiology; however, a substantial proportion of patients had hormonally active or malignant tumors, reflecting the complexity of referrals to a tertiary endocrine center.

Our findings highlight the importance of systematic hormonal evaluation and careful radiological characterization. In addition, a multidisciplinary approach remains essential to optimize management decisions, avoid unnecessary interventions, and ensure appropriate long-term follow-up. This study provides valuable regional data and supports the implementation of international recommendations within a North African healthcare context.
